# Supplementary material for: Elevated risk of invasive group A streptococcal disease and host genetic variation in the human leucocyte antigen locus
Source: Genes Immun. 2019 Aug 29;21(1):63–70. doi: 10.1038/s41435-019-0082-z (PMC7039814; doi:10.1038/s41435-019-0082-z)
Supplement: Supplementary file 1 — Supplementary Material. [file 41435_2019_82_MOESM1_ESM.pdf]

Supplementary Table 1: Quality Control of Genome-wide Genotyping Data

Process	Step	Threshold for exclusion	Removed	Carried Forward
Individual QC	Missingness	Missingness > 5%	1 case<br>7 controls	49 cases<br>2054 controls
	Heterozygosity	Outliers removed<br>(see Supplementary Figure 3)	2 cases<br>46 controls	47 cases<br>2008 controls
	Relatedness	Relatedness > 0.094	1 case<br>399 controls	46 cases<br>1609 controls
	Ancestry	Non-European outlier removed<br>(see Supplementary Figure 4)	3 cases<br>69 controls	43 cases<br>1540 controls
Variant QC	Missingness	Missingness > 0.01	73 variants	119251 variants
	Frequency	Minor allele frequency < 0.05	85 variants	119166 variants
	Hardy-Weinberg	Hardy-Weinberg *P*-value < 10^{-10}	9 variants	119157 variants
	Two Locus Test[32]	Outlier removed<br>(see Supplementary Figure 5)	23 variants	119134 variants

Supplementary Table 2: Association statistics for imputed HLA alleles associated with susceptibility

**Allele**	**MAF**	**Imputation R^{2}***	**OR (95% CI)**	P_{LMM}
*HLA-DRB1**13:01	0.057	0.988	2.19 (1.13-4.23)	0.020
*HLA-DQA1**01:03	0.061	0.991	2.31 (1.23-4.36)	0.009
*HLA-DQB1**06:03	0.061	0.936	2.25 (1.18-4.31)	0.014

MAF, minor allele frequency; OR, odds ratio; CI, confidence intervals; LMM, linear mixed-model.
*Beagle software[34] (v3.0.4) R^2 statistic

Supplementary Table 3: Association statistics for imputed HLA amino acids associated with susceptibility

Gene	IMGT Position	Amino acid	Direction	MAF	Imputation R^{2}*	OR (95% CI)	P_{LMM}
HLA-A	9	Tyrosine	Presence	0.150	0.994	0.44 (0.20-0.96)	0.041
HLA-C	35	Glutamine	Presence	0.136	0.999	1.86 (1.11-3.11)	0.018
HLA-C	138	Lysine	Presence	0.136	0.997	1.85 (1.10-3.10)	0.020
HLA-C	156	Leucine-Arginine	Absence	0.234	0.995	0.51 (0.27-0.94)	0.031
HLA-C	275	Glycine	Presence	0.136	0.996	1.86 (1.11-3.11)	0.018
HLA-DQA1	41	Lysine	Presence	0.061	0.990	2.32 (1.23-4.37)	0.009
HLA-DQA1	130	Alanine	Presence	0.061	0.990	2.32 (1.23-4.36)	0.009
HLA-DPB1	84	Glycine	Absence	0.315	0.976	1.55 (1.01-2.38)	0.043
HLA-DPB1	205	Valine	Absence	0.124	0.766	2.01 (1.14-3.53)	0.015
HLA-DPB1	215	Isoleucine	Absence	0.111	0.748	1.97 (1.09-3.55)	0.024

IMGT, International Immunogenetics Information System; MAF, minor allele frequency; OR, odds ratio; CI, confidence intervals; LMM, linear mixed-model.
*Beagle software[34] (v3.0.4) R^2 statistic

Supplementary Table 4: Association statistics for imputed previously implicated HLA haplotypes

**Haplotype**	**Direction***	**MAF**	**Info†**	**OR (95% CI)**	P_{LMM}
*DRB1**15:01-*DQA1**01:02-*DQB1**06:02	Protective	0.158	1.0	0.87 (0.47-1.64)	0.67
*DRB1**03:01-*DQA1**05:01-*DQB1**02:01	Protective	0.142	1.0	0.97 (0.52-1.82)	0.93
*DRB1**14:01-*DQA1**01:01-*DQB1**05:03	Predisposition	0.0262	1.0	2.36 (0.94-5.91)	0.067
*DRB1**11:01-*DQA1**05:01-*DQB1**03:01	Predisposition	0.0753	1.0	0.79 (0.25-2.54)	0.70
*DRB1**07:01-*DQA1**03:01-*DQB1**02:01	Predisposition	0	-	-	-

MAF, minor allele frequency; OR, odds ratio; CI, confidence intervals; LMM, linear mixed-model.
*Reported in Kotb et al. 2002[4]
†SNPTEST software (v2.5.4) Info statistic

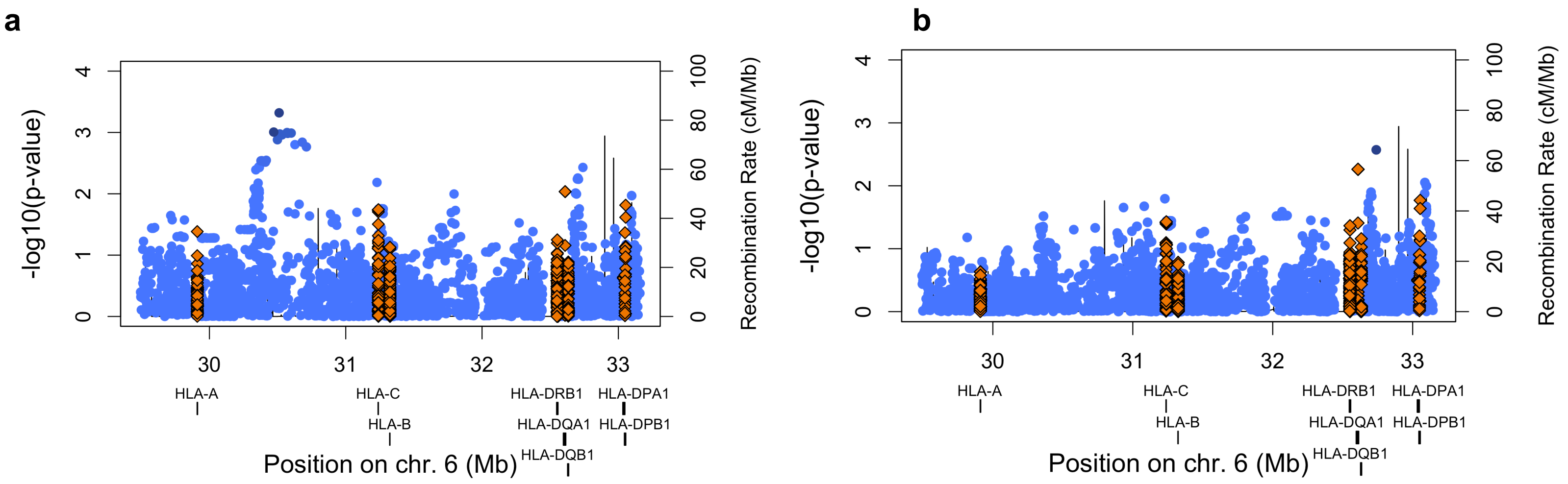

Supplementary Figure 1: Association signals across the histocompatibility complex. Regional association plots are shown with genomic position plotted against the negative common logarithm of the p-value from a linear mixed-model analysis **(a)** before and **(b)** after conditioning on rs2524222. Amino acids are indicated by gold-coloured diamonds while SNPs are indicated blue circles with the depth of the blue proportional to the degree of linkage disequilibrium with the most associated variant. The recombination rate is shown as a line plotted on the right-hand y-axis.

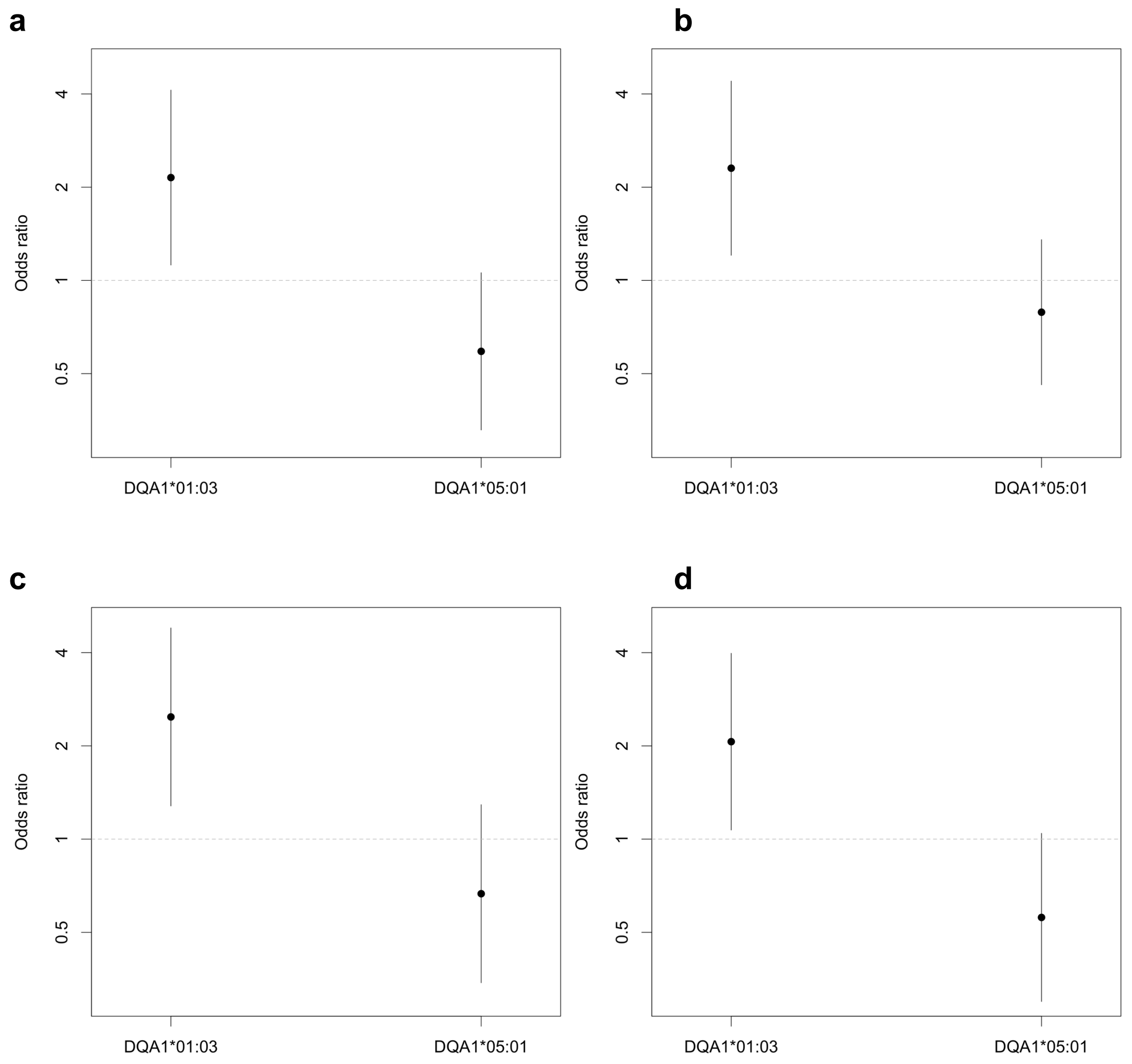
a
b
c
d
Odds ratio
4
2
1
0.5
DQA1*01:03
DQA1*05:01

Supplementary Figure 2: Effect size estimates for *HLA-DQA101:03 using alternative analytical approaches.** Effect size estimates with confidence intervals are plotted for *HLA-DQA1**01:03 and *HLA-DQA1**05:01 based on a model including parameters for both alleles and sex using **(a)** a linear mixed-model with transformation[36], **(b)** logistic regression with no additional parameters, **(c)** logistic regression with parameters for the first ten principal components[10], and **(d)** a generalized linear mixed-model[11].

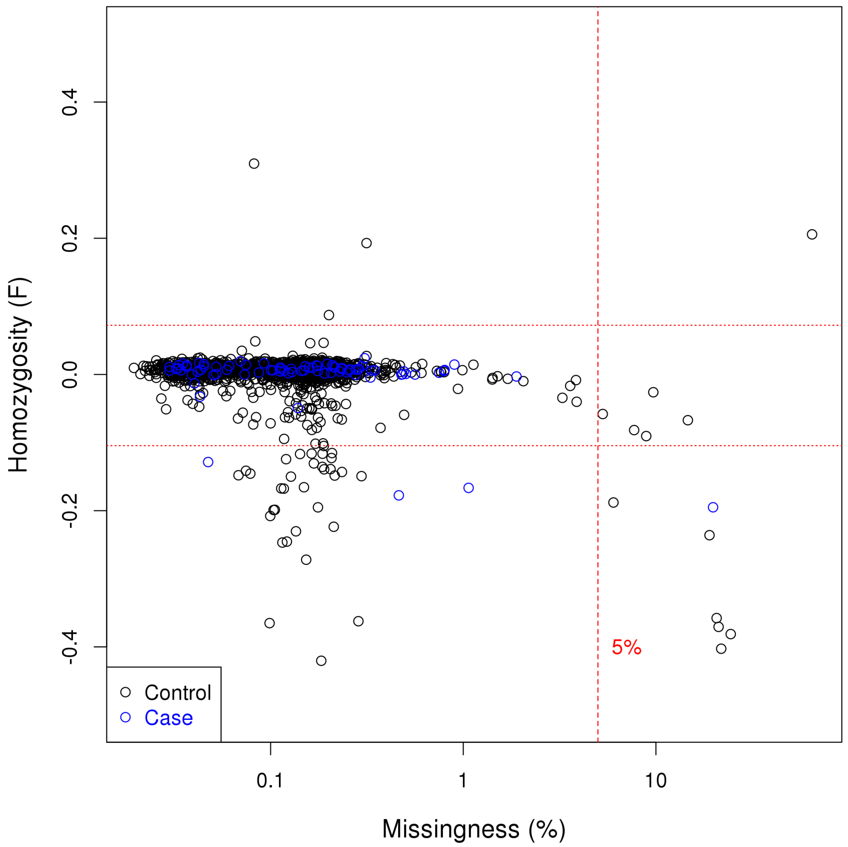

Supplementary Figure 3: Assessment of heterogeneity and missingness in the study. Autosomal homozygosity is plotted against missingness on a logarithmic scale. Horizontal lines are drawn at two standard deviations above and three below the mean of autosomal homozygosity with missingness less than 5%.

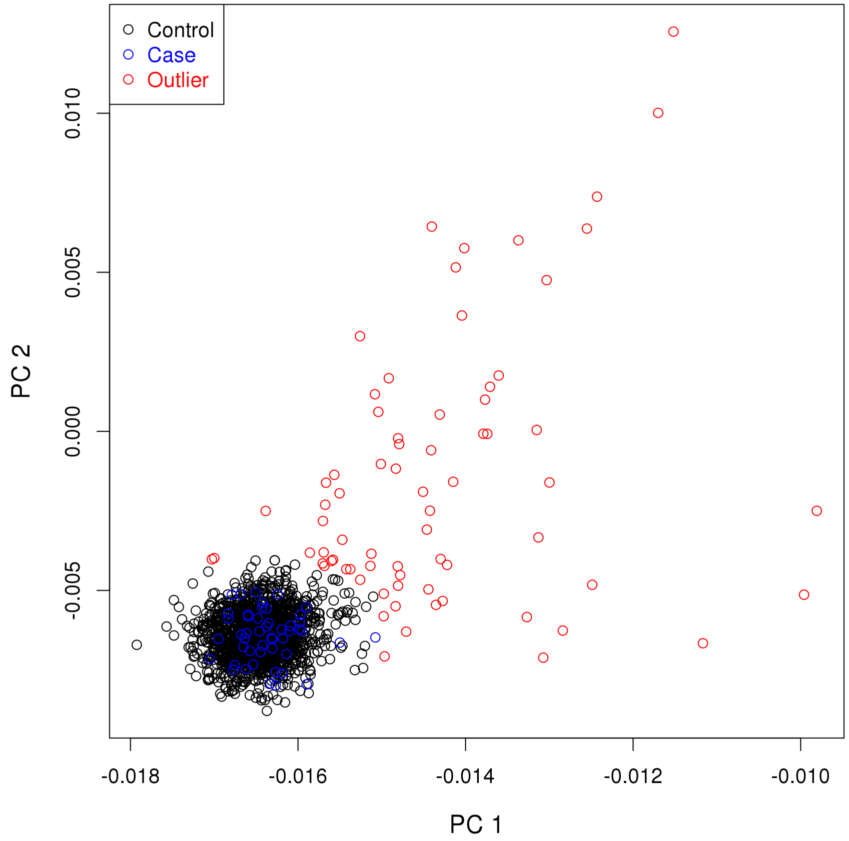

Supplementary Figure 4: Principal component analysis of ancestry to definite European ancestry. Principal components analysis was run with individuals of African and East Asian ancestry from the HapMap consortium data and outlying samples removed based on distance from the British European cluster.

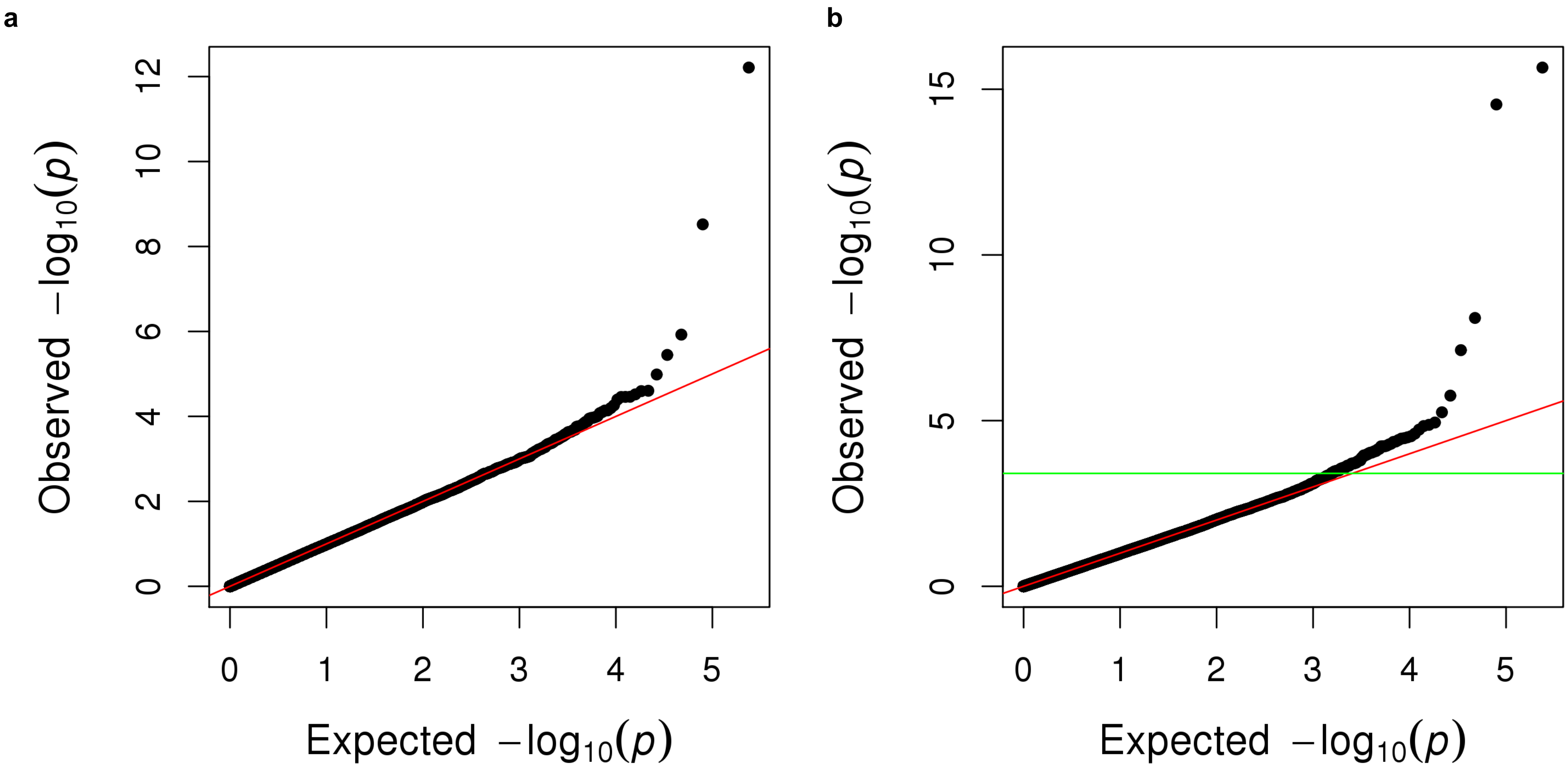

Supplementary Figure 5: Removal of variants by the two locus quality control test. Quantile-quantile plots are shown for genome-wide susceptibility analysis performed with **(a)** unadjusted linear regression and **(b)** the genome-wide linear model-based quality control test. In the latter, the horizontal green line is drawn at the point at which the negative common logarithm of the observed *P*-value exceeds the expected value by greater than 0.2 (based on Lee *et al.*[32]). Variants were excluded when the test statistic calculated in both directions exceeded this threshold.
